# Dietary glycyrrhizin enhances reproductive performance by improving intestinal microbiota, liver lipid metabolism and ovarian senescence in aged breeder hens

**DOI:** 10.1186/s40104-025-01288-5

**Published:** 2025-11-20

**Authors:** Zhenwu Huang, Huchuan Liu, Guangju Wang, Huan Ge, Yanru Shi, Jinghai Feng, Chunmei Li, Minhong Zhang

**Affiliations:** 1 State Key Laboratory of Animal Nutrition and Feeding, Institute of Animal Science, Chinese Academy of Agricultural Sciences, No. 2 Yuanmingyuan West Road, Haidian District, Beijing, 100193 China; 2 College of Animal Science and Technology, Nanjing Agricultural University, Nanjing, 210095 China; 3 Qingdao Animal Husbandry and Veterinary Research Institute, Qingdao, 266100 China

**Keywords:** Aged breeder hen, Glycyrrhizin, Gut microbiota, Lipid metabolism, Reproductive performance

## Abstract

**Background:**

The decline in reproductive performance of aged hens is mainly attributed to oxidative damage in reproductive organs, hepatic lipid metabolism disorders, and intestinal microbiota dysbiosis. Glycyrrhizin (GL) has been proven to enhance antioxidant capacity, regulate lipid metabolism and gut microbiota in mammals, but its efficacy in hens remains unclear. Hence, this study aimed to investigate whether dietary GL supplementation improves reproductive performance in hens during the late laying stage by modulating intestinal microbiota composition, hepatic lipid metabolism and ovarian antioxidant status.

**Results:**

Dietary supplementation with 100 mg/kg GL significantly improved the egg production rate, egg quality, and hatching rate in aged breeder hens (*P* < 0.05). GL supplementation also increased the serum levels of HDL-C, TP and ALB, and enhanced the antioxidant capacity in both serum and ovary (*P* < 0.05). In addition, dietary GL elevated the serum progesterone (P4) levels by enhancing the transcription level of steroid synthesis key enzymes (*CYP11A1* and *3β-HSD*) in the ovary (*P* < 0.05). Dietary GL also promoted the synthesis and transport of vitellogenin (VTG) by upregulating the *VTG-II* (*P* < 0.05) and *APOV1* (*P* = 0.077) expression levels in the liver, thereby increasing the number of grade follicles and small yellow follicles. Moreover, dietary GL enhanced hepatic fatty acid β-oxidation by upregulating *PPARα* and *CPT-I* (*P* < 0.05), and downregulating *ACC* expression levels (*P* < 0.05). In agreement, liver metabolomics analysis revealed that dietary GL supplementation significantly altered hepatic metabolism, with 389 differentially identified metabolites (*P* < 0.05). The key metabolites (e.g., taurocholic acid, tauroursodeoxycholic acid, nicotinuric acid, glycodeoxycholic acid (hydrate)) were identified, and they were mainly functionally enriched in beta-alanine metabolism nicotinate, taurine and hypotaurine metabolism (*P* < 0.05). Finally, 16S rRNA gene sequencing revealed that dietary GL reversed age-induced changes in gut microbiota composition, characterized by a significant increase in *Lactobacillus* abundance and a decrease in *Bacteroides* (*P* < 0.05).

**Conclusions:**

These results collectively demonstrate that dietary supplementation with 100 mg/kg GL improved reproductive performance by reversing age-induced changes in gut microbiota, enhancing hepatic vitellogenin synthesis, and ameliorating ovarian function in aged breeder hens. This study suggests that dietary GL is a potential strategy to improve reproductive performance in broiler breeder hens during the late laying period.

**Graphical Abstract:**

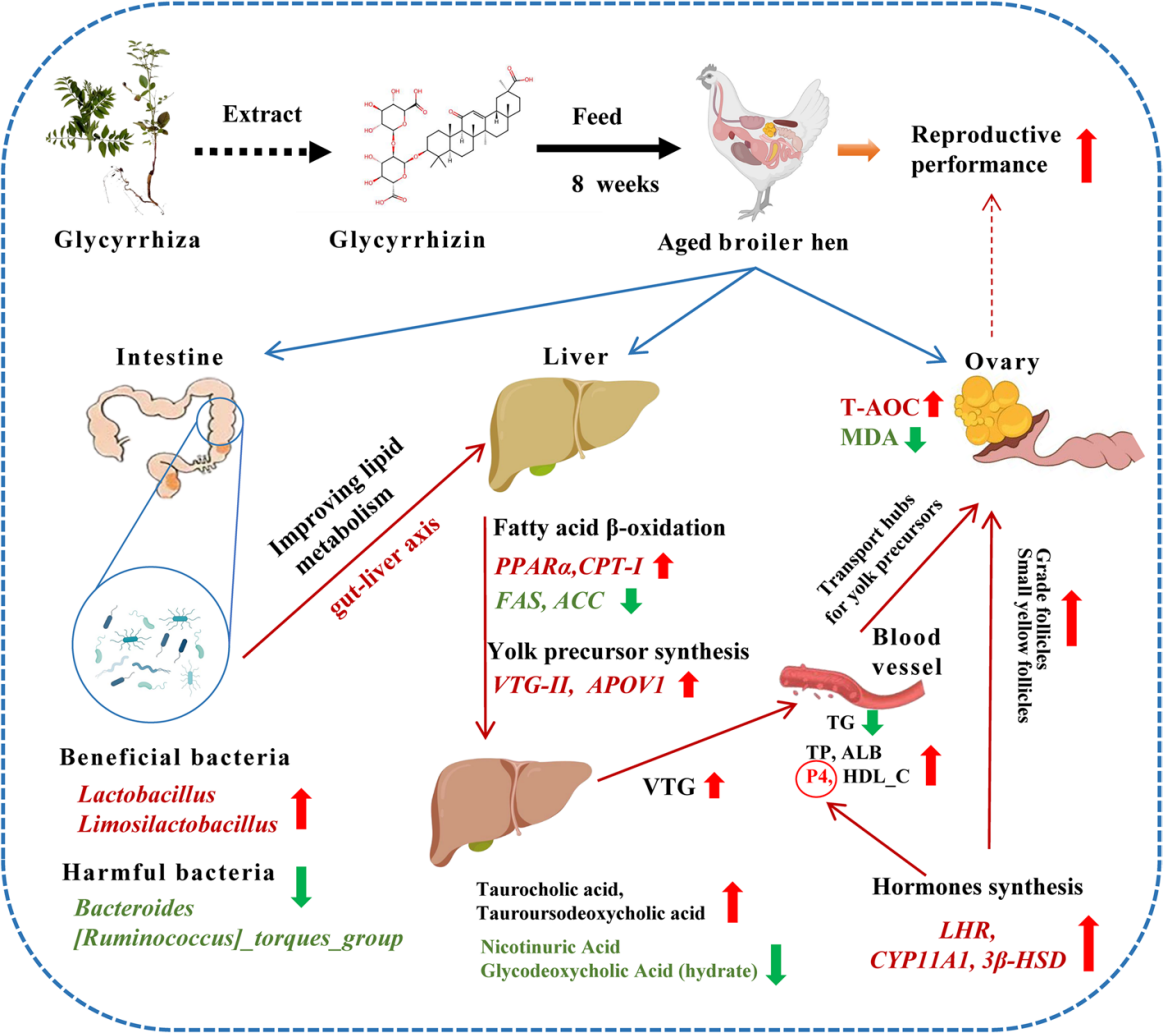

**Supplementary Information:**

The online version contains supplementary material available at 10.1186/s40104-025-01288-5.

## Background

In the late laying phase, both egg production and hatchability of broiler breeders decline sharply, resulting in substantial economic losses. The decline of reproduction performance in aging hens is a complex process involving functional deterioration across multiple systems, which is generally attributed to intestinal dysfunction [1], dysregulation of hepatic lipid metabolism [2, 3] and ovarian senescence [4]. With advancing age, ovarian senescence is closely associated with oxidative damage resulting from the accumulation of reactive oxygen species (ROS) [4]. Excessive oxidative accumulation directly leads to follicular atresia, which disrupts steroid production in granulosa cells and reduces the number of developing follicles [4]. Previous studies have proved that the serum levels of steroid hormones estradiol (E2) and progesterone (P4) are significantly lower in aged hens [5–7]. Unlike in mammals, the avian liver serves as the primary site not only for lipid metabolism but also for the synthesis of egg yolk precursors, which are transported via the blood to developing oocytes [8]. Consequently, hepatic synthesis of yolk precursors critically regulates follicular maturation and subsequent egg production. In commercial caged laying hens, fatty liver syndrome is a prevalent metabolic disease during the late laying phase, resulting from prolonged egg-laying stress and confinement [3, 9]. Numerous studies have shown that the typical characteristics of fatty liver syndrome include excessive fat deposition in the liver, hepatic steatosis, impaired fat metabolism, and reduced synthesis of yolk precursors [3, 10, 11]. This inevitably disturbs yolk deposition and follicle maturation, leading to a significant decrease in egg production. Furthermore, numerous investigations have revealed that the composition and abundance of gut microorganisms are strongly associated with the reproductive performance of hens [12–14]. This is thought to be related to the involvement of microorganisms in regulating the host's metabolism and the immune activity [11, 12, 15]. It has been reported that Bacteroidetes replace Firmicutes as the dominant phylum in aged hens [16]. Aging-induced reductions in the abundance of beneficial intestinal microbiota represent a critical cause contributing to the decline in egg production rate and egg quality [17, 18]. In order to improve the egg-laying rate of late-phase laying hens, several strategies, including probiotics [19], organic acids [20, 21], enzyme preparations [22], and active plant ingredients [4, 8, 23, 24], have been extensively investigated. However, there were still a few studies focusing on the nutritional regulation of reproductive performance in aging breeders. Therefore, it is of great necessity to explore the efficacy and safety of additives and their potential mechanisms of action in aged breeders, particularly broiler breeders, as these are thought to help prolong their productive life.

Glycyrrhiza is a well-known traditional Chinese medicinal plant that is widely used and listed in the pharmacopoeias of many Asian countries [25]. Recent studies have demonstrated that glycyrrhiza extracts have significant effects in promoting animal growth, improving oxidative status and regulating lipid metabolism [26–28]. Toson et al. [28] found that dietary glycyrrhiza extracts improved the growth performance, serum lipid metabolism and antioxidant capacity in chicks. Glycyrrhizin (GL), also referred to glycyrrhizic acid, is a major active ingredient of glycyrrhiza and exhibits antimicrobial, antioxidant, anti-inflammatory, and antihyperlipidemic properties in mammals [29–31]. It has been reported that GL ameliorates oxidative stress and mitochondrial damage while reducing the production of inflammatory factors in neonatal rats [32]. Its antioxidant activity occurs primarily through Nrf2 activation and free radical scavenging [33]. Furthermore, GL is recognized for its efficacy in liver protection and regulation of hepatic lipid metabolism [34]. Wang et al. [35] reported that glycyrrhizinate (pharmaceutical form of GL) has a good effect on alleviating alcohol-induced liver injury in mice by reducing oxidative damage and disorder of fat. A similar study has confirmed that GL ameliorates liver injury through improving abnormalities in bile acid metabolism in obese mice [34]. Notably, hepatic bile acid metabolism is closely associated with gut microbiota composition, especially *Lactobacillus* spp. [36]. The study by Xu et al. [37] reported that combined supplementation with GL and probiotics enhanced gut microbiota homeostasis and accelerated growth performance in piglets by enriching *Lactobacillus* spp. Interestingly, a recent study demonstrated that dietary GL enhanced the antioxidant capacity of broilers, mitigating the inflammatory response and apoptosis caused by zearalenone in the glandular stomach [38]. These studies indicate that GL plays an important role in enhancing antioxidant capacity, regulating lipid metabolism and modulating gut microbiota in animals. Here, we hypothesize that dietary GL supplementation increases beneficial gut bacteria, enhances hepatic lipid metabolism, and alleviates ovarian senescence via reducing oxidative stress, thereby improving reproductive performance in aged broiler breeder hens.

Thus, the present study aims to investigate the effects of GL on the reproduction performance of aged broiler breeder hens and to elucidate its underlying molecular mechanisms in multiple organs. Our findings provide insights for developing nutritional strategies for aging broiler breeder hens and expand the potential applications of GL in poultry production.

## Materials and methods

### Test material and basic diet preparation

The glycyrrhizin (GL) monomer with more than 98% purity was purchased from Tianfeng Biological Co., Ltd. (Xi'an, China). The licorice roots were processed through sorting, washing, drying, and slicing. The processed licorice was then extracted using ethanol as the solvent. Ultimately, glycyrrhizin (CAS: 1405-86-3) was obtained through concentration, purification, and drying, resulting in a white crystalline powder. The basic diet used in this study was referred to the nutrient requirement of the China National Feeding Standard of Chicken (NY/T33-2004) (Table 1). During the trial period, 80 kg of feed for the GL group was freshly prepared every 4 d. Specifically, 2 kg of basal feed was thoroughly mixed with 8 g of GL, and then this premix was blended into the remaining 78 kg of feed and mixed thoroughly. Additionally, the components of the trial diets were analyzed based on our previously reported method [39].

**Table 1:** Ingredient compositions and nutrient levels of the basic corn-soybean diet

Ingredient	Content, %	Nutrient level^2^	Content
Corn	62.48	Avian metabolic energy, MJ/kg	11.48
Soybean meal	23.40	Crude protein, %	15.94
Limestone	8.70	Available phosphorus, %	0.51
Soybean oil	2.10	Methionine, %	0.49
Dicalcium phosphate	2.01	Lys, %	0.80
NaCl	0.35	Thr, %	0.58
Vitamin-mineral premix^1^	0.62		
Choline chloride (50%)	0.12		
DL-Methionine	0.22		
Total	100.00		

^1^The premix (per kilogram of diet) provided the following: vitamin A, 12,500 IU; vitamin D_3_, 2,500 IU; vitamin E, 30 mg; vitamin K_3_, 2.60 mg; thiamine, 2 mg; riboflavin, 6 mg; vitamin B_12_, 0.025 mg; biotin, 0.0325 mg; folic acid, 1.25 mg; pantothenic acid, 12 mg; niacin, 50 mg Cu, 8 mg; Zn, 75 mg; Fe, 80 mg; Mn, 100 mg; Se, 0.2 mg; I, 0.35 mg

^2^Nutrient levels were calculated values

### Animals and treatments

A total of 192 aged broiler breeder hens (Hubbard efficiency plus breeder hens, 50-week-old) were randomly divided into two treatment groups: (1) a basic corn-soybean diet (CON); (2) the basic corn-soybean diet supplemented with 100 mg/kg glycyrrhizin. Each group contained 8 replications, with 12 hens per replicate. The supplemental GL level in this study was chosen according to a previous study [38]. The hens were raised in an environmentally controlled house at the Jiulian 10th Breeding Farm (Qingdao, China) using battery cages. Hens from 6 adjacent cages on one tier were considered an experimental replicate, and different replicates were separated by baffles. After one week of recording and adjusting the laying rate, all groups were found to have similar egg-laying rates. The formal experiment lasted for 8 weeks. Each hen was restricted fed 146 g/d at 04:30 throughout the experimental period. Water was provided ad libitum via nipple drinkers. The light regimen was 16L:8D (light:dark). The indoor temperature and humidity were maintained at 20–23 °C and 50%–60%, respectively. Breeder roosters (42-week-old) were raised in the same room as hens, with a male-to-female ratio of 1:10. During the experiment, hens were artificially inseminated with 20 μL of pooled semen by experienced stockmen every 4 d. After collection, the semen was stored in the water at 37 °C, with insemination completed within 30 min.

### Reproductive performance and egg quality measurement

The number and weight of eggs were recorded daily to evaluate the laying performance throughout the experimental period. In the final week of the experiment, the breeder eggs with uniform coloration, intact shells (free of spots or cracks), and weighing between 62 and 68 g were selected to evaluate fertility and hatchability. The 204 breeder eggs from per group were collected and hatched in a commercial incubator (2112 type incubator, Limin Comp., Dezhou, China). Each treatment contained 6 replicates of 34 breeder eggs. The incubation process was strictly followed according to manufacturer guidelines, and the mode of chicken (Table S1) was set. On d 8 of incubation, all hatching eggs were candled and unfertilized eggs were removed. After hatching, the number of hatched chicks and healthy chicks was recorded, respectively. The healthy chick rate (%) = (the number of healthy chicks/the total number of chicks) × 100%, and the fertilized eggs hatchability (%) = (the number of newly hatched chicks/the number of fertilized eggs) × 100%. The calculation formulas for feed conversion ratio (FCR), fertilization rate and hatchability were described in our previous study [2]. At the end of weeks 4 and 8 of the experiment, 16 eggs from each treatment (2 eggs per replicate) were randomly collected for the determination of egg quality. Egg weight, albumen height, Haugh unit, and yolk color were immediately measured by the digital analyzer (Robotmation Co., Place, Japan). The yolk and albumen were then separated using a yolk separator and individually weighed to determine the relative yolk and albumen proportion.

### Sample collection

At the end of the official experiment, all hens were individually weighed, and one hen from each replicate (with body weight close to the group average) was randomly selected. According to the record throughout the production period, egg-laying time for breeder hens was around 11:00 h. Therefore, the sample collection was commenced at 08:00 h. To minimize interference from the laying cycle on the experimental results, all treated hens were sampled at the same stage of their cycle. After reweighing, blood samples were collected from the wing vein of all hens within 30 min, and serum was separated by centrifugation (3,000 × *g*, 15 min, 4 °C) followed by storage at −80 °C for subsequent analyses. Hens were euthanized via cervical dislocation, the liver, spleen, abdominal adipose tissue, and ovaries were immediately collected and weighed. Liver samples were collected from the right lobe, while ileal contents were collected from the mid-ileum. Ovaries were completely removed from the abdominal cavity, photographed and the follicles were visually counted. Subsequently, small yellow follicles (SYF), liver tissues, and ileal contents were stored at −80 °C for subsequent analysis. An additional liver tissue sample (approximately 1 cm^2^) was excised and fixed in 4% paraformaldehyde (PFA) for morphological analysis. Follicles were classified into 3 types based on size and color: grade follicles (> 10 mm), small yellow follicles (4 to 10 mm), and large white follicles (2 to 4 mm) according to a previously reported method [40].

### Biochemical assays of liver and serum

Serum biochemical indicators, including the total cholesterol (T-CHO), low density lipoprotein cholesterol (LDL-C), high density lipoprotein cholesterol (HDL-C), triglyceride (TG), total protein (TP), albumin (ALB), alkaline phosphatase (ALP), alanine aminotransferase (ALT), aspartate aminotransferase (AST) and uric acid (UA) were measured using commercial kits (Nanjing Jiancheng Bioengineering Institute, Nanjing, China). The concentrations of serum E2 and P4 were assayed using commercial ELISA kits (Nanjing Jiancheng Bioengineering Institute, Nanjing, China). Hepatic vitellogenin (VTG) levels in the liver were assayed using a VTG ELISA kit (Baililai Biotechnology Co., Ltd., Shanghai, China). All procedure steps were performed according to the manufacturer's instructions and the reading was performed with a Power Wave XS2 microplate reader (BioTek, Winooski, VT, USA).

### Antioxidant assays of ovary and serum

Approximately 200 mg of ovarian tissues were homogenized in cold 0.9% saline and centrifuged to obtain the tissue homogenate supernatant. The levels of total antioxidant capacity (T-AOC), catalase (CAT) activity, total superoxide dismutase (T-SOD) activity, and malondialdehyde (MDA) levels in both ovarian tissue and serum were determined using commercial kits (Nanjing Jiancheng Bioengineering Institute, Nanjing, China). The total protein content in the ovarian tissue homogenate supernatant was quantified with an Enhanced BCA Protein Assay Kit (Beyotime Biotechnology, Shanghai, China). All ovarian measurements were normalized to the protein concentration.

### Morphology analysis of liver

After fixation in 4% PFA for 24 h, liver samples were progressively dehydrated with ethanol and xylene and then embedded with paraffin. Sections of 5 μm thickness were prepared using a Leica RM2235 rotary microtome (Leica Microsystems, Wetzlar, Germany). The sections were deparaffinized in xylene and rehydrated through a descending ethanol gradient. Subsequently, staining was performed with hematoxylin and eosin (HE) (Sevier Biological Technology Co., Ltd., Wuhan, China). Finally, images were taken using a DM68 Leica bright-field/fluorescence microscope (Leica, Wetzlar, Germany).

### Total RNA extraction and mRNA quantification

Total RNA was extracted from liver and ovary tissue using the FastPure Cell/Tissue total RNA isolation kit V2 (Vazyme Biotechnology, Nanjing, China) according to the kit instructions. Subsequently, total RNA concentration and quality were assessed using an ND-2000 microspectrophotometer (Thermo Scientific, Wilmington, USA). A total of 1,000 ng RNA was reverse-transcribed into complementary DNA using the HiScript II First Strand cDNA synthesis kit (Vazyme Biotechnology, Nanjing, China). After synthesis, the cDNA was diluted 1:4 with RNase-free water. Quantitative real-time PCR (qRT-PCR) was performed using a QuantStudio 7 Flex system (Thermo Fisher Scientific, USA) with ChamQ Universal SYBR qPCR Master Mix (Vazyme Biotech, Nanjing, China). The primer sequences were synthesized by Sangon Biotechnology (Beijing, China) and showed in Table S2. The relative expression of the mRNA of the target gene was analyzed by the ${2}^{-\Delta \Delta \text{Ct}}$ method after normalization against *GAPDH*.

### Sequencing of the 16S ribosomal RNA (rRNA) gene

Six ileal content samples per group were randomly selected for 16S ribosomal RNA (rRNA) gene sequencing analysis. The genomic DNA was extracted using the CATB method, and the V3–V4 region of 16S rRNA genes was amplified by the specific primer with the barcode (341F, 5'-CCTAYGGGRBGCASCAG-3'; 806R, 5'-GGACTACNNGGGTATCTAAT-3'). Phusion High-Fidelity PCR Master Mix (New England Biolabs, Ipswich, MA, USA) was used to perform the PCR reactions. Sequencing libraries were generated using the NEBNext Ultra II DNA Library Prep Kit (New England BioLabs, Ipswich, MA, USA). Finally, the library was sequenced on the Illumina NovaSeq 6000 platform (Illumina, San Diego, CA, USA). All steps mentioned above were conducted by the Novogene Co., Ltd. (Beijing, China) and bioinformatic analyses were performed on the Novogene Cloud Platform (https://magic.novogene.com). The specific steps of visualization and analysis were referred to the methods of earlier literature [41].

### Untargeted metabolic analysis of liver

Six liver samples per group were randomly selected for untargeted metabolome analysis. The metabolomic profiling analysis included sample preparation, metabolite separation and detection, quality control, and metabolomic data preprocessing. Briefly, samples were homogenized and extracted with methanol, then split into aliquots for analysis by ultrahigh-performance liquid chromatography/mass spectrometry (UHPLC-MS/MS) in both positive and negative polarity modes. The Orbitrap Q Exactive HF-X mass spectrometer (Thermo Fisher) and the Vanquish UHPLC system (Thermo Fisher) were used in UHPLC-MS/MS. Raw data files generated by UHPLC-MS/MS were processed using Compound Discoverer 3.1 to perform peak alignment, peak picking, and quantitation for each metabolite. Peaks were matched with the mzCloud (https://www.mzcloud.org/), mzVault, and MassList databases to obtain the accurate qualitative and relative quantitative results. These metabolites were annotated using the KEGG database, HMDB database, and LIPIDMaps database. The software R (R version R-3.4.3), Python (Python 2.7.6 version), and CentOS (CentOS release 6.6) were used to perform statistical analyses. The Novogene Cloud Platform (https://magic.novogene.com) was used for the final analysis and visualization of all data.

Multivariate methods, including PCA and PLS-DA, were used for normalized data analysis. Differentially abundant metabolites were identified based on the following parameters: *P* < 0.05 (Student’s *t*-test) and variable importance in projection (VIP) > 1. Metabolites that reached these criteria were marked as significantly differential metabolites. Significantly differential metabolites obtained from each comparison group were mapped to KEGG identifiers and subsequently used for relevant pathway analysis on the KEGG website.

### Statistical analysis

Data were analyzed using SPSS (version 26.0; SPSS Inc., Chicago, IL, USA) with Student’s independent samples *t*-test. Results are expressed as mean ± standard error of the mean (SEM). Pearson correlations were used to examine the relationship between different variables. A *P*-value < 0.05 was considered statistically significant, and 0.05 < *P* < 0.10 was considered to be a trend towards significance.

## Results

### Effects of dietary GL on reproductive performance and relative organ weight of aged hens

**Fig. 1:**
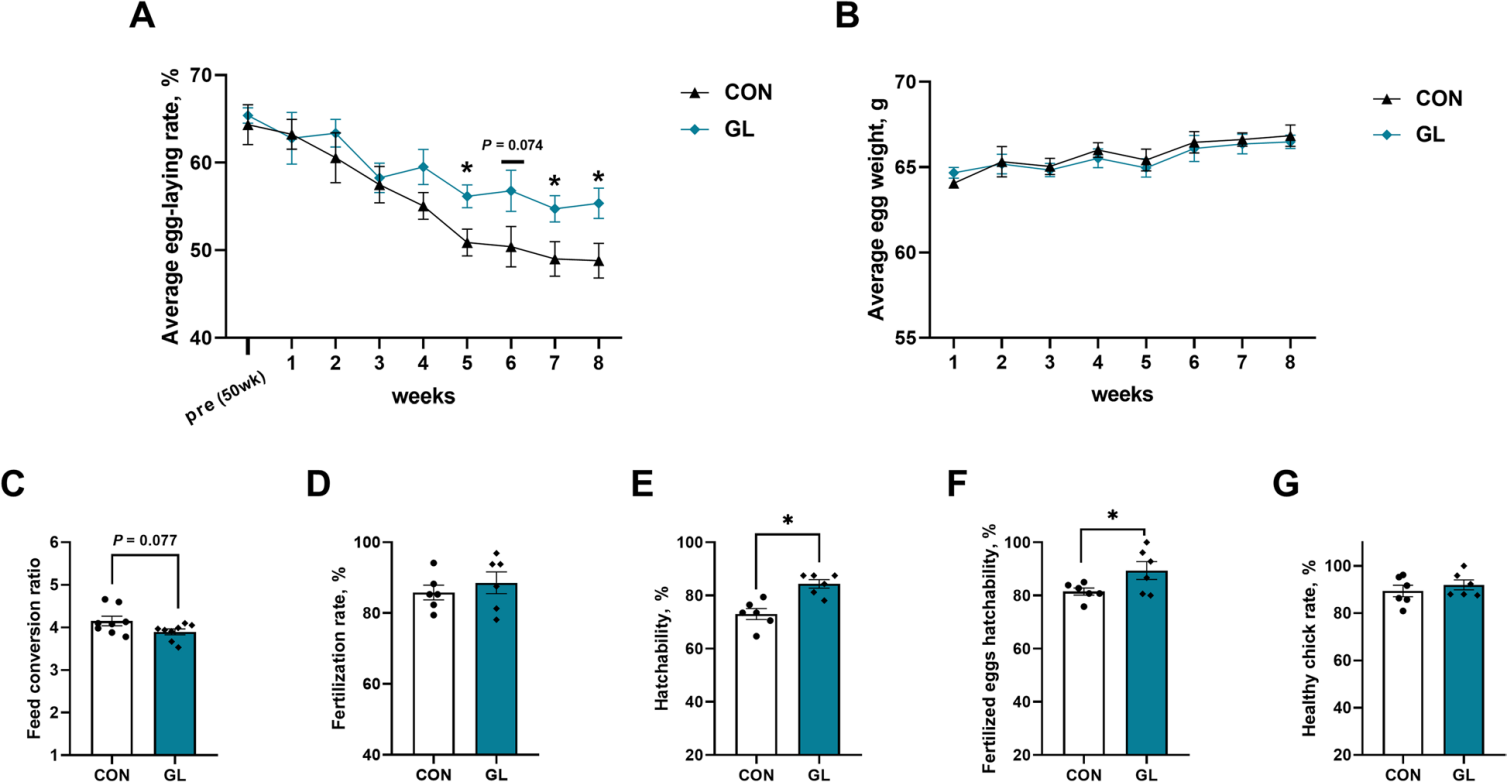
Effects of dietary glycyrrhizin on reproductive performance of aged hens. **A** Average egg-laying rate (*n* = 8). **B** Average egg weight (*n* = 8). **C** Feed conversion rate (*n* = 8). **D** Fertilization rate (*n* = 6). **E** Hatchability (*n* = 6). **F** Fertilized eggs hatchability (*n* = 6). **G** Healthy chick rate. CON represents hens fed with basal diet; GL represents hens fed with a basic diet supplemented with 100 mg/kg glycyrrhizin. Asterisks indicate significant differences between groups (*P* < 0.05)

To assess the effects of GL supplementation on reproductive performance of aged breeder hens, we analyzed the laying rate, hatching performance and feed efficiency (Fig. 1). Dietary GL significantly increased egg production rate at weeks 5, 7 and 8 compared with the CON group (*P* < 0.05, Fig. 1A). While, there was no statistically difference in average egg weight between the two groups throughout the experimental period (Fig. 1B). The feed conversion ratio (FCR) showed a decreasing trend in the GL group (*P* = 0.077, Fig. 1C). Moreover, hatchability and the fertilized eggs hatchability significantly higher in the GL group than in the CON group (*P* < 0.05, Fig. 1E and F). No significant differences were observed in fertilization rate and healthy chick rate between the groups (Fig. 1D and G). In addition, as shown in Table S3, dietary GL tended to increase relative abdominal fat weight (*P* = 0.066). There were no significant differences in the relative organ weight of liver, spleen, and ovary between the groups.

### Effects of dietary GL on egg quality of aged hens

As shown in Table 2, dietary GL significantly altered egg quality characteristics. At week 8, dietary supplementation with GL exhibited a significant increase in albumen height (*P* < 0.05) and a tendency toward higher Haugh units (*P* = 0.083). However, there was no significant difference in the egg weight, yolk weight, albumen weight, yolk ratio, and yolk color throughout the experimental period.

**Table 2:** Effects of dietary glycyrrhizin supplementation on egg quality of aged hens

Items	Groups		*P*-value
	CON	GL	
4 weeks			
Egg weight, g	67.2 ± 3.2	68.4 ± 2.9	0.642
Yolk weight, g	21.6 ± 0.4	21.3 ± 0.5	0.557
Albumen weight, g	32.7 ± 2.0	34.3 ± 2.1	0.116
Yolk ratio, %	32.3 ± 2.5	31.6 ± 1.8	0.354
Haugh unit	84.7 ± 1.4	83.9 ± 2.1	0.450
Albumen height, mm	7.51 ± 0.31	7.89 ± 0.33	0.596
Yolk color	6.55 ± 0.39	6.79 ± 0.33	0.305
8 weeks			
Egg weight, g	67.4 ± 3.8	66.9 ± 3.7	0.266
Yolk weight, g	21.9 ± 0.5	21.5 ± 0.4	0.532
Albumen weight, g	35.7 ± 1.9	35.6 ± 2.8	0.893
Yolk rate, %	32.0 ± 0.55	32.1 ± 0.55	0.932
Haugh unit	94.2 ± 4.3	97.6 ± 4.1	0.083
Albumen height, mm	8.74 ± 0.51	9.90 ± 0.22*	0.009
Yolk color	7.65 ± 0.12	7.76 ± 0.17	0.567

CON represents hens fed with basal diet; GL represents hens fed with a basic diet supplemented with 100 mg/kg glycyrrhizin. Data are expressed as mean ± SEM (*n* = 16). Asterisks indicate significant differences between groups (*P* < 0.05)

### Effects of dietary GL on serum biochemical parameters and reproductive hormone levels of aged hens

The effects of dietary GL on serum biochemical parameters of aged hens are represented in Table 3. Compared with the CON group, GL supplementation significantly increased serum concentrations of TP and ALB (*P* < 0.05). In addition, GL supplementation showed a tendency to increase serum HDL_C levels (*P* = 0.079) and reduce TG concentrations (*P* = 0.082). No significant differences were observed in other serum biochemical parameters between the two groups. Serum steroid hormone assessment revealed that GL treatment significantly increased progesterone levels (*P* < 0.05) but had no significant effect on estradiol concentration.

**Table 3:** Effects of dietary glycyrrhizin supplementation on the serum reproductive hormone levels and biochemical indicators of aged breeder hens

Items	Groups		*P*-value
	CON	GL	
Reproductive hormones			
E2, ng/mL	237.0 ± 17.0	201.3 ± 17.1	0.682
P4, ng/L	41.0 ± 4.8	57.7 ± 4.5*	0.024
Biochemical indicators			
LDL_C, mmol/L	3.15 ± 0.14	2.93 ± 0.19	0.373
HDL_C, mmol/L	1.72 ± 0.22	2.38 ± 0.27	0.079
TG, mmol/L	14.4 ± 1.4	10.2 ± 1.7	0.082
T-CHO, mmol/L	7.41 ± 0.43	6.59 ± 0.19	0.273
TP, g/L	59.9 ± 7.8	74.1 ± 12.7*	0.016
ALB, g/L	18.9 ± 1.5	20.8 ± 1.7*	0.036
ALT, U/L	10.9 ± 4.3	9.7 ± 2.1	0.475
AST, U/L	82.6 ± 6.7	79.0 ± 7.9	0.732
UA, μmol/L	201.7 ± 70.4	224.0 ± 12.4	0.667

CON represents hens fed with basal diet; GL represents hens fed with a basic diet supplemented with 100 mg/kg glycyrrhizin. Data are expressed as mean ± SEM (*n* = 8). Asterisks indicate significant differences between groups (*P* < 0.05)

### Effects of dietary GL on oxidative status in aged hens

The effects of dietary GL on the oxidative status of the serum and ovary in aged hens are presented in Table 4. Dietary GL supplementation significantly increased total antioxidant capacity in both serum and the ovary (*P* < 0.05). Besides, MDA content in serum and ovary was also diminished compared to the control group (*P* < 0.05). Whereas there was no significant difference in the activity of T-SOD and CAT between the two groups.

**Table 4:** Effects of dietary glycyrrhizin supplementation on oxidative status of serum and ovary in aged breeder hens

Items	Groups		*P*-value
	CON	GL	
Serum			
T-AOC, U/mL	34.6 ± 8.5	42.3 ± 2.6*	0.028
T-SOD, U/mL	14.4 ± 0.88	13.9 ± 0.40	0.273
CAT, U/mL	50.6 ± 9.9	46.1 ± 4.9	0.306
MDA, nmol/mL	9.29 ± 3.87	5.79 ± 1.31*	0.030
Ovary			
T-AOC, U/mg prot	24.5 ± 3.6	34.5 ± 4.1*	0.019
T-SOD, U/mg prot	97.6 ± 11.2	107.5 ± 9.4	0.653
CAT, U/mg prot	6.74 ± 1.43	7.69 ± 1.67	0.465
MDA, nmol/mg prot	5.31 ± 0.96	3.24 ± 1.84*	0.021

CON represents hens fed with basal diet; GL represents hens fed with a basic diet supplemented with 100 mg/kg glycyrrhizin. Data are expressed as mean ± SEM (*n* = 8). Asterisks indicate significant differences between groups (*P* < 0.05)

### Effects of dietary GL on follicle development and expression of steroid synthesis genes in SYF

**Fig. 2:**
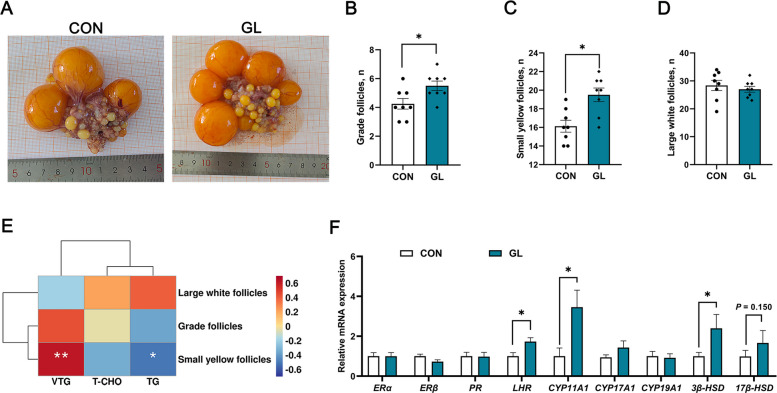
Effects of dietary glycyrrhizin on follicle development in the ovary of aged hens (*n* = 8). **A** Typical macroscopic morphology of ovary. **B**–**D** The number of grade follicle, small yellow follicles, and large white follicles. **E** Heat map of Pearson’s correlations between hepatic lipid metabolism and grade follicles. **F** Expression of genes related to steroid receptors and steroidogenic enzymes in the ovary. CON represents hens fed with basal diet; GL represents hens fed with a basic diet supplemented with 100 mg/kg glycyrrhizin. Asterisks indicate significant differences between groups (*P* < 0.05); Double asterisk indicates extremely significant difference (*P* < 0.01)

Figure 2A shows representative macroscopic appearances of the ovaries. Compared with the control group, dietary GL supplementation significantly increased the numbers of grade follicles (GF) and small yellow follicles (SYF) in aged hens (*P* < 0.05, Fig. 2B and C), but not significantly altered the numbers of the large white follicles (LWF) (Fig. 2D). Notably, a significant positive Pearson correlation was observed between the numbers of small yellow follicles and liver VTG contents, while a significant negative correlation was found with liver TG contents (Fig. 2E). Regarding ovarian reproduction-related gene expressions, GL treatment significantly up-regulated the mRNA expressions of luteinizing hormone receptor (*LHR*), cytochrome P450 family 11 subfamily A member 1 (*CYP11A1*), and 3 β-hydroxysteroid dehydrogenase (*3β-HSD*) in the ovary of aged hens (*P* < 0.05, Fig. 2F). There was no different on the mRNA expressions of estrogen receptor alpha and beta (*ERα*, *ERβ*), progesterone receptor (*PR*), cytochrome P450 family 17/19 subfamily A member 1 (*CYP17A1*, *CYP19A1*), and 17 β-hydroxysteroid dehydrogenase (*17β-HSD*) in the ovary between two groups.

### Effects of dietary GL on lipid metabolism and yolk precursor synthesis in liver

**Fig. 3:**
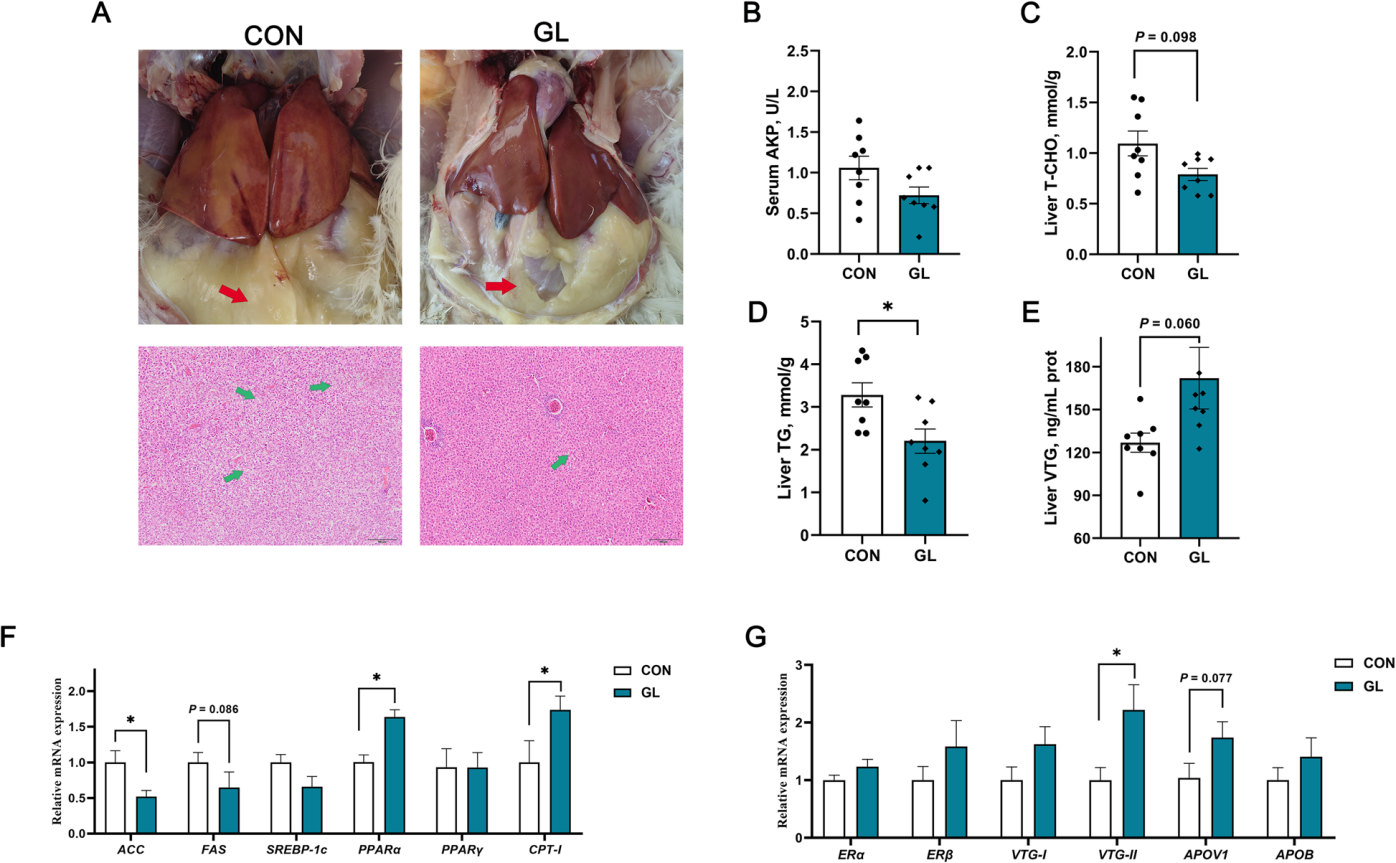
Effects of dietary glycyrrhizin on lipid metabolism and yolk precursor synthesis in the liver of aged hens (*n* = 8). **A** Typical macroscopic morphology and histological analysis of liver. **B**–**E** The levels of AKP, T-CHO, TG and VTG in the liver. **F** Expression of genes related to lipid metabolism in the liver. **G** Expression of genes related to yolk precursor in the liver. Scale bar = 100 μm. Red arrows point at abdominal fat, green arrows point at lipid vacuoles. CON represents hens fed with basal diet; GL represents hens fed with a basic diet supplemented with 100 mg/kg glycyrrhizin. Asterisks indicate significant differences between groups (*P* < 0.05)

The liver serves as the primary site of yolk precursor synthesis, which is closely associated with the reproductive performance of laying hens. Macroscopic morphology and HE staining observation of the liver was conducted for intuitively examining tissue injury of aged hens (Fig. 3A). Compared with the CON group, the liver from GL treatment exhibited bright red coloration and no hepatic steatosis overall. Additionally, H&E staining further indicated reduced lipid vacuolation in the GL treatment. Consistent with histological findings showing reduced lipid vacuoles, GL treatment significantly decreased the liver TG levels in aged hens (*P* < 0.05, Fig. 3D). A decreasing trend was also observed in T-CHO levels (*P* = 0.096, Fig. 3C), while vitellogenin (VTG) levels showed an increasing trend (*P* = 0.060, Fig. 3E). The expression of key genes related to lipid metabolism and yolk precursor synthesis was further analyzed (Fig. 3F and G). The relative gene expression of peroxisome proliferator-activated receptor alpha (*PPARα*), carnitine palmitoyltransferase-I (*CPT-I*), and vitellogenin-II (*VTG-II*) in the liver of the GL group was significantly upregulated (*P* < 0.05). However, the gene expression of *ACC* was significantly downregulated in the GL group (*P* < 0.05). The expression of *FAS* exhibited a marked downward trend (*P* = 0.086), whereas apovitellenin-1 (*APOV1*) showed an upward trend (*P* = 0.077). No significant differences were observed in the expression of sterol regulatory element-binding protein 1c (*SREBP-1c*), peroxisome proliferator-activated receptor gamma (*PPARγ*), *ERα, ERβ,* vitellogenin-I (*VTG-I*), and apolipoprotein B (*APOB*) between the two groups.

### Effects of dietary GL on liver metabolomics profile in aged hens

**Fig. 4:**
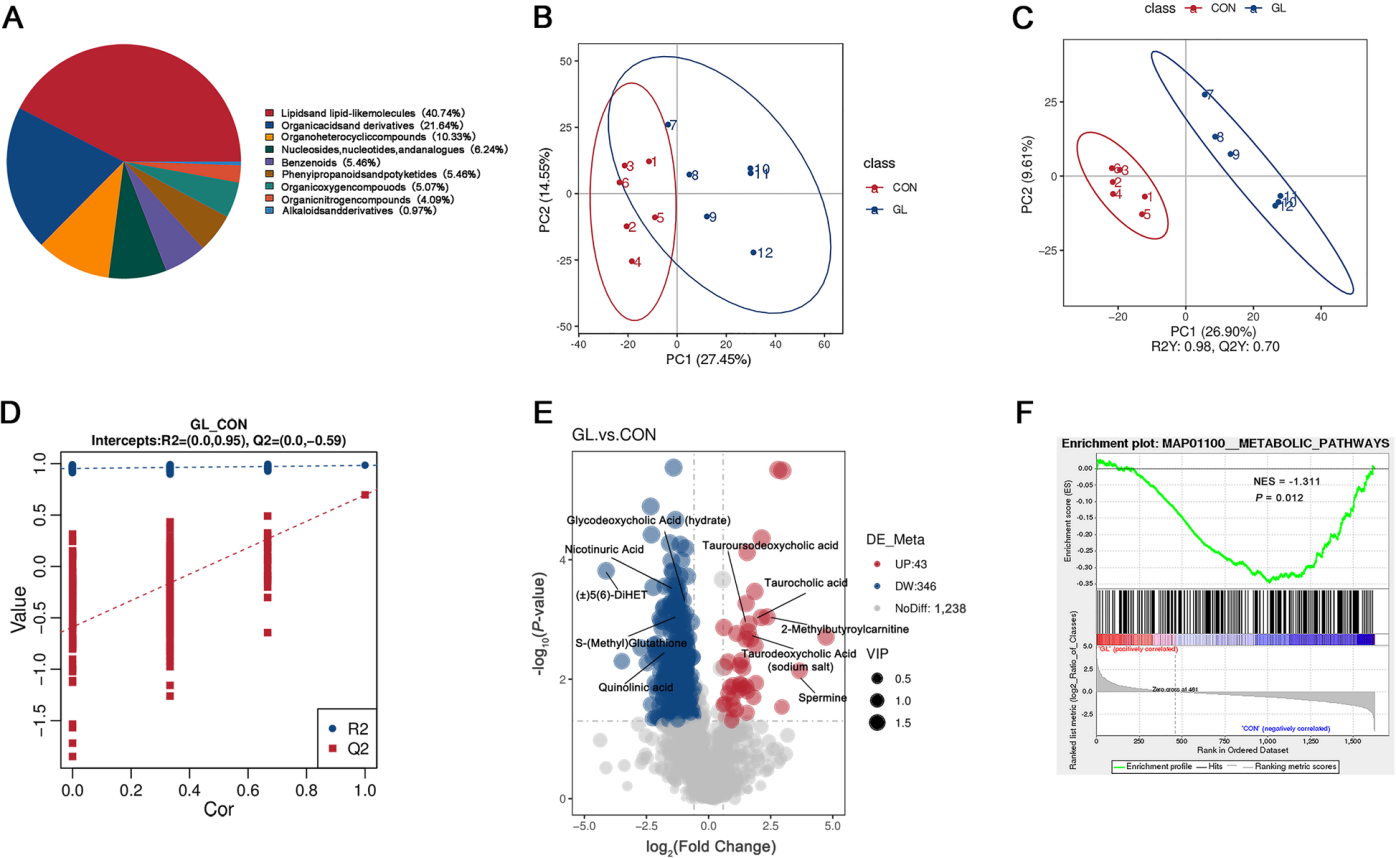
Effects of dietary glycyrrhizin on the hepatic metabolome in the liver of aged hens (*n* = 6). **A** Superclass pie chart of differential metabolites in the liver. **B** PCA plots of total samples. **C** PLS-DA scores of total samples. **D** Plot of the permutation test of PLS-DA modes. **E** Volcano plot of differential metabolites. **F** GSEA enrichment analysis associated metabolism pathway. CON represents hens fed with basal diet; GL represents hens fed with a basic diet supplemented with 100 mg/kg glycyrrhizin

**Fig. 5:**
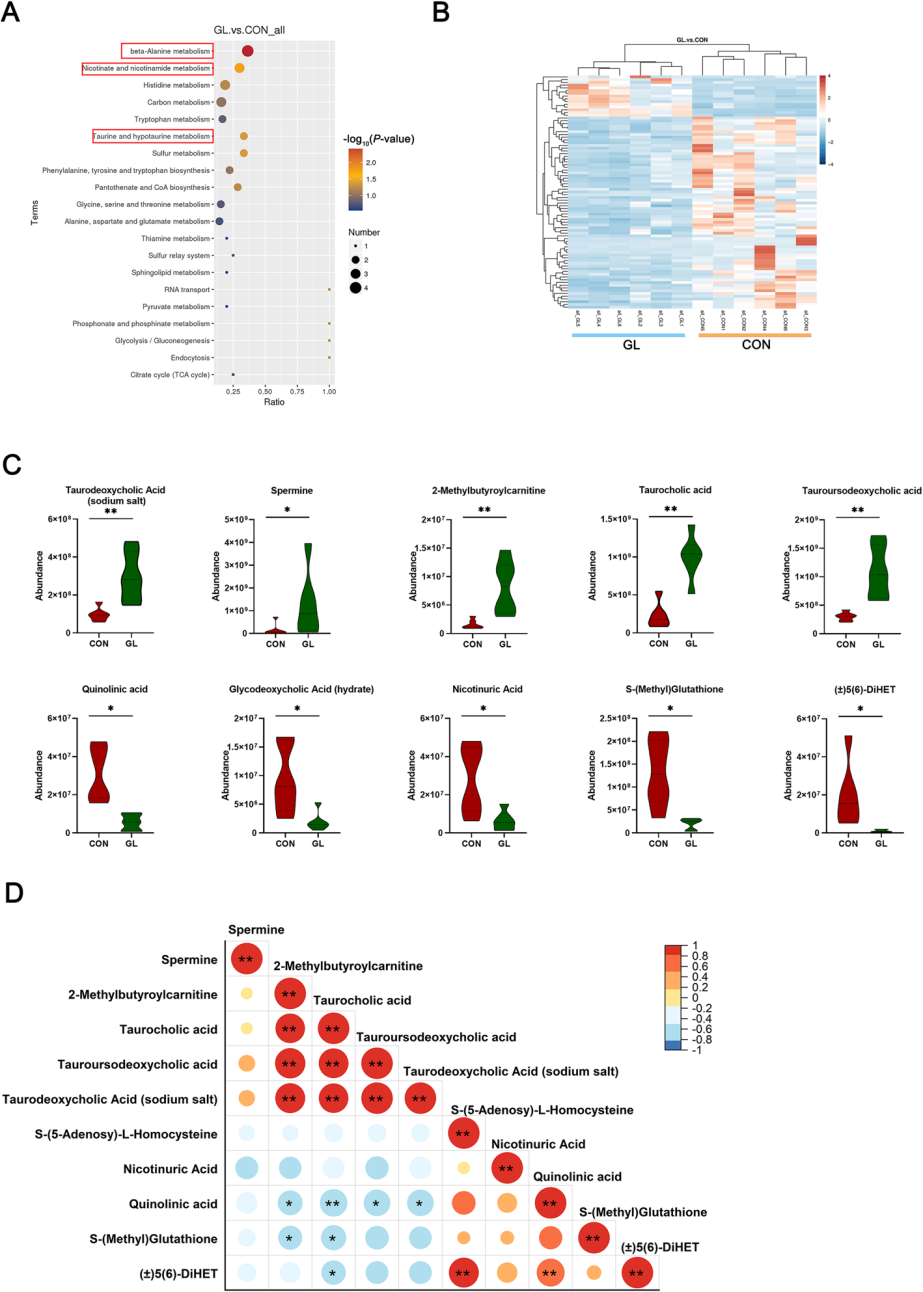
Metabolomic analysis in the liver of aged hens (*n* = 6). **A** The differential metabolites related top 20 of the enriched KEGG pathways. **B** The cluster heatmap showing the key up-regulated and down-regulated differential metabolites between CON group and GL group. **C** The differential marker metabolite contents between CON group and GL group. **D** Heat map of Pearson’s correlations for the 10 key marker differential metabolites. CON represents hens fed with basal diet; GL represents hens fed with a basic diet supplemented with 100 mg/kg glycyrrhizin. Asterisks indicate significant differences between groups (*P* < 0.05); Double asterisk indicates extremely significant difference (*P* < 0.01)

To investigate the potential mechanism by which glycyrrhizin modulates hepatic lipid metabolism in aged hens, the liver untargeted metabolome analysis was conducted. The PCA plot showed that clear separation of metabolites between CON and GL groups (Fig. 4B). Pie chart analysis based on metabolite SuperClass indicated that the majority of differentially abundant metabolites were lipids and lipid-like molecules, organic acids and derivatives and organoheterocyclic compounds (Fig. 4A). Afterward, significant differences in metabolites between CON and GL groups were further assessed using PLS-DA (Fig. 4C). The results showed markedly separated clusters between two groups, and the permutation test with 200 iterations proved that the OPLS-DA models were not overfitted (*R*^2^ = 0.95 and Q^2^ = −0.59, Fig. 4D). A total of 389 metabolites were considered as differential metabolites according to the threshold (*P* < 0.05 and VIP > 1), with volcano plots indicating 43 significantly upregulated and 346 significantly downregulated metabolites (Fig. 4E). Gene set enrichment analysis (GSEA) of all differential metabolites revealed significant downregulation of hepatic metabolic pathways (Fig. 4F), suggesting substantial alterations in lipid metabolism consistent with histological observations of reduced hepatic fat vacuolization. In order to identify the key differential metabolites, the thresholds (*P* < 0.05 and |log_2_ FC| > 3) were used to further screen for differential metabolites. The cluster heatmap showed high reproducibility of key metabolites within the same treatment group and significant variation between different groups (Fig. 5B). Furthermore, pathway analysis using the KEGG database identified 30 enriched metabolic pathways, with the top 20 shown in Fig. 5A, including beta-alanine metabolism, nicotinate and nicotinamide metabolism, and taurine and hypotaurine metabolism. Ten differential metabolites associated with these pathways were identified as key regulators. As shown in Fig. 5C and D, these key metabolites include spermine, 2-methylbutyroylcarnitine, taurocholic acid, tauroursodeoxycholic acid, taurodeoxycholic acid (sodium salt), nicotinuric acid, glycodeoxycholic acid (hydrate), quinolinic acid, S-(methyl) glutathione, and (±)5(6)-DiHET.

### Correlation of key differential metabolites and biochemical parameters

**Fig. 6:**
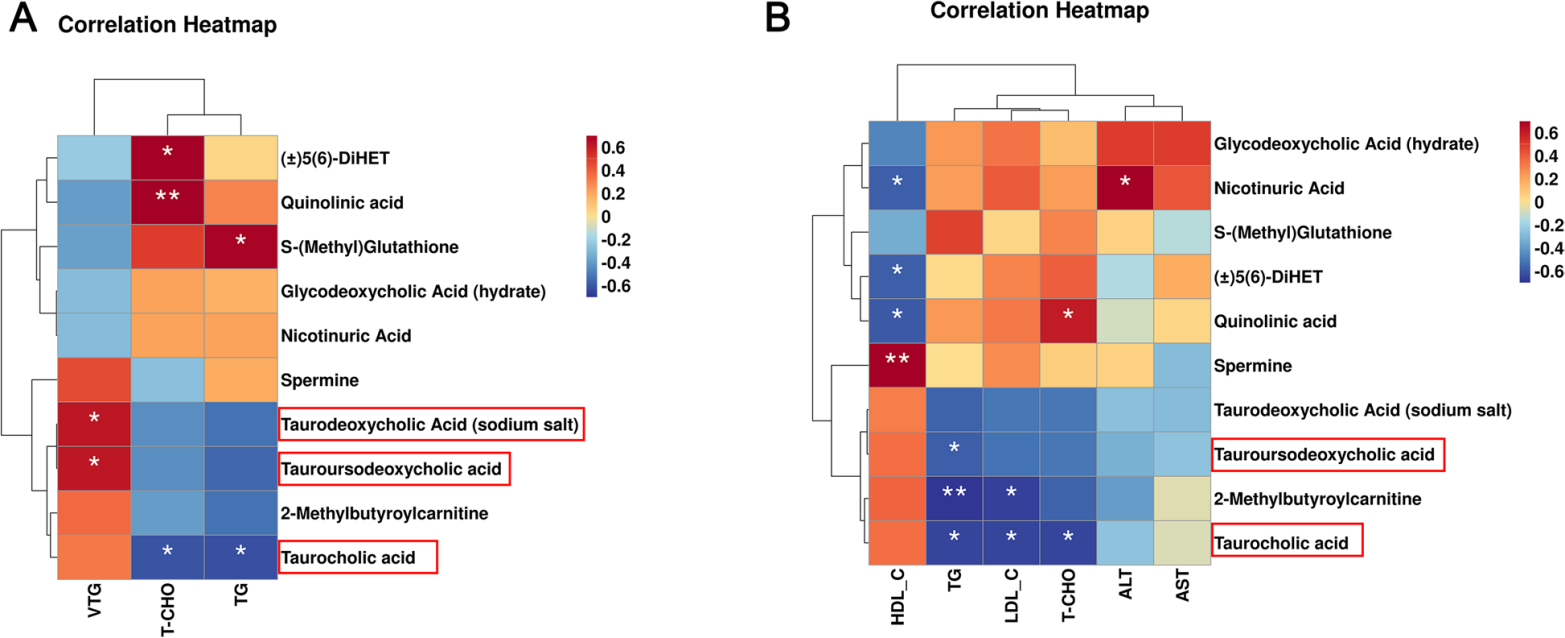
Correlation analysis of differential metabolites (*n* = 6). **A** A heatmap of Pearson’s correlation between top 10 key differential metabolites and liver biochemical parameters. **B** A heatmap of Pearson’s correlation between top 10 key differential metabolites and serum biochemical parameters. Asterisks indicate significant differences between groups (*P* < 0.05); Double asterisk indicates extremely significant difference (*P* < 0.01)

To explore the role of 10 key metabolites in hepatic lipid metabolism and function in aged hens, Pearson's correlation analysis was conducted to examine their relationship with associations with biochemical parameters in both liver and serum. As illustrated in Fig. 6, tauroursodeoxycholic acid and taurodeoxycholic acid (sodium salt) showed a significant positive correlation with hepatic vitellogenin (VTG) content (*P* < 0.05), suggesting that taurine and hypotaurine metabolism are closely associated with vitellogenin synthesis in the liver. In addition, taurocholic acid levels were significantly negatively correlated with T-CHO and TG contents in the liver and serum (*P* < 0.05). Quinolinic acid and (±)5(6)-DiHET levels were significantly positively correlated with hepatic T-CHO (*P* < 0.05), and negatively correlated with serum HDL_C (*P* < 0.05).

### Structure of the intestinal microbiota in aged hens

**Fig. 7:**
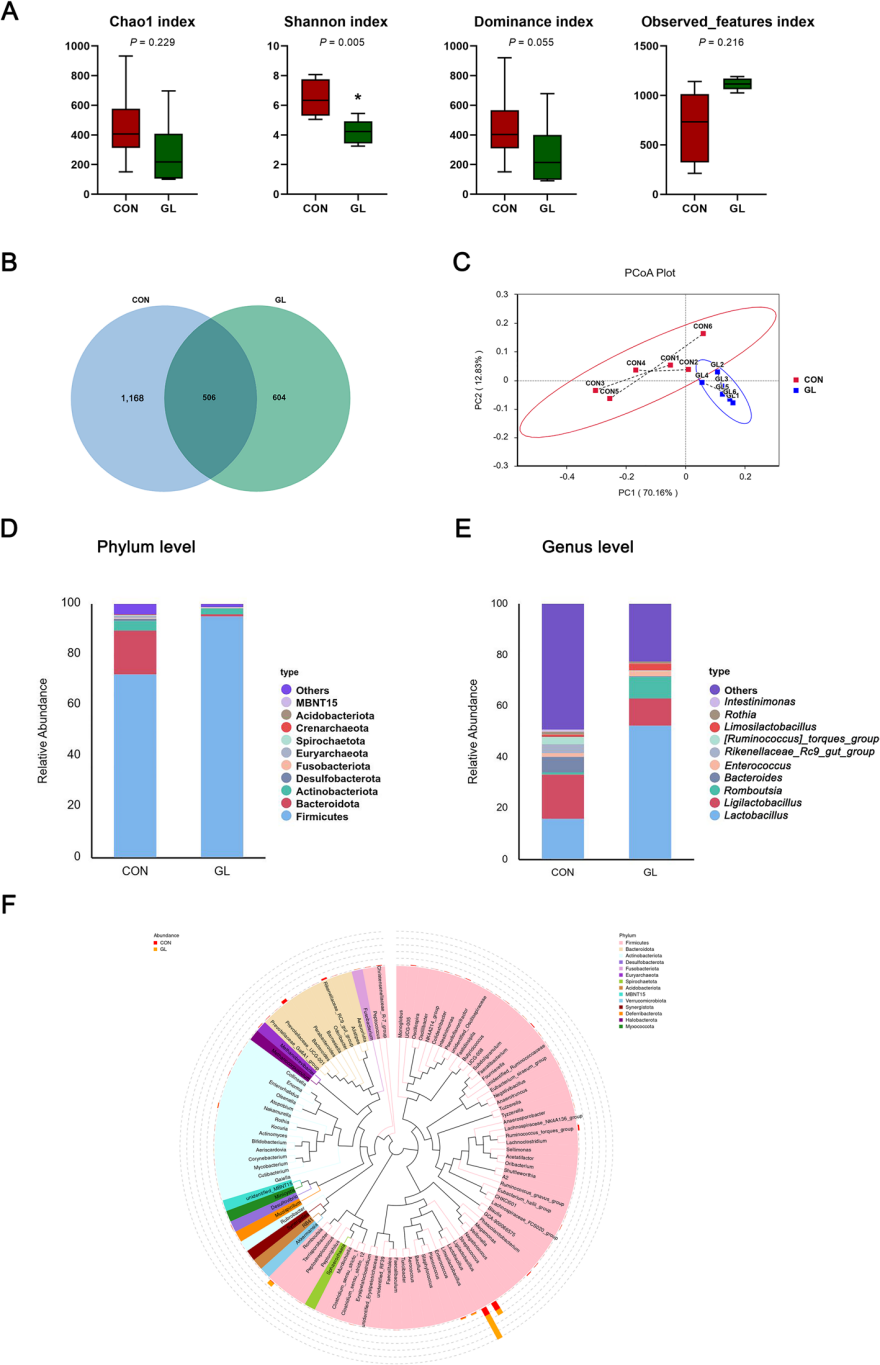
Effects of dietary GL on ileal content microbial communities of aged hens. **A** Alpha diversity analysis (*n* = 6). **B** Venn diagram of OTUs level. **C** Principal coordinate analysis (PCoA) scores plot of the samples. **D** and **E** Relative abundance of the bacteria at the phylum and genus. **F** Enriched taxa represented in cladograms. CON represents hens fed with basal diet; GL represents hens fed with a basic diet supplemented with 100 mg/kg glycyrrhizin. Asterisks indicate significant differences between groups (*P* < 0.05)

**Fig. 8:**
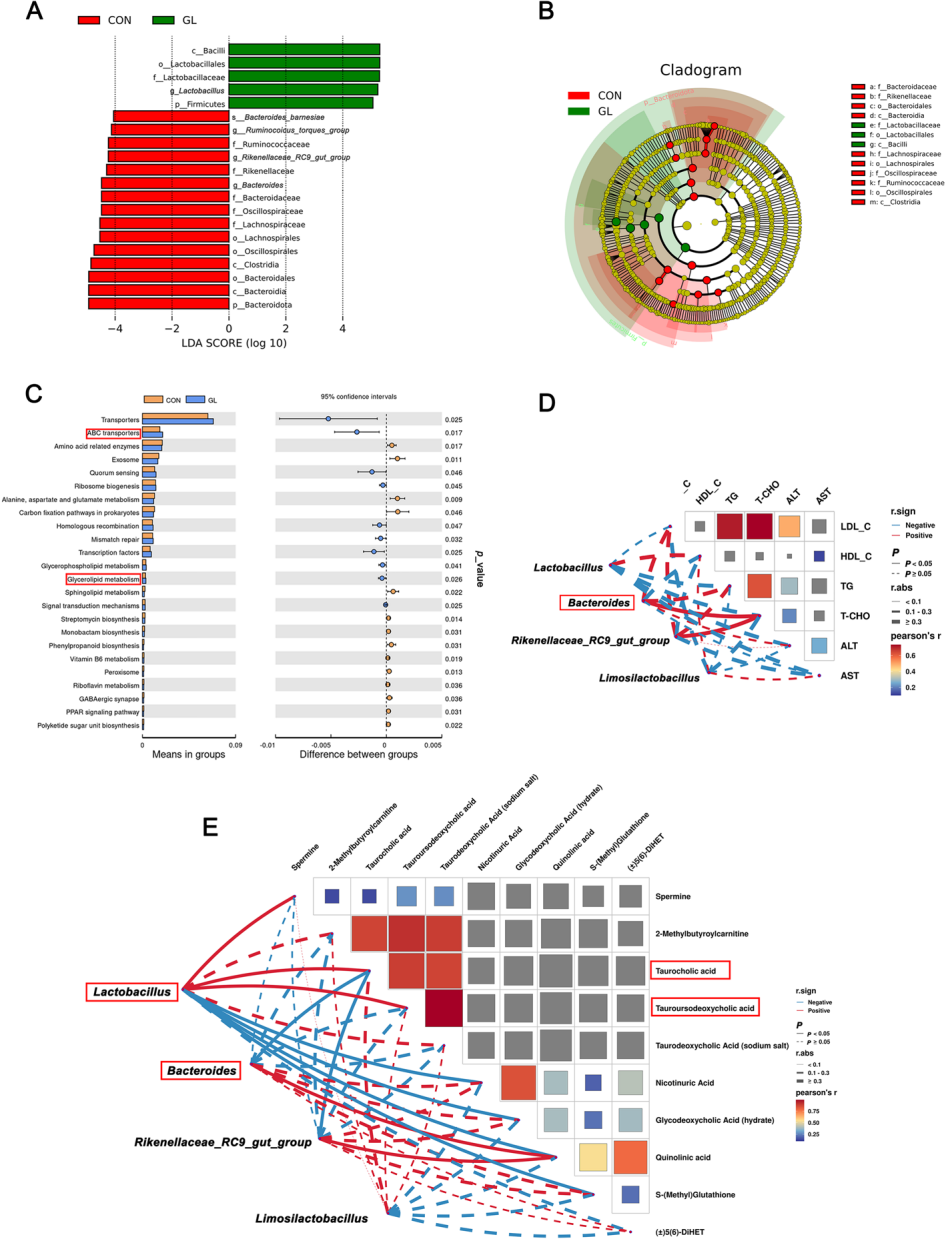
Effects of dietary GL on ileal content microbial structures and correlation analysis with differential metabolites (*n* = 6). **A** Linear discriminant analysis (LDA) score distribution. **B** Cladogram plot of the biomarkers. **C** Predictive functional profiling of microbial communities by Tax4Fun analysis (level 3 of KEGG). **D** Mantel test assessing associations between serum biochemical parameters and key bacteria abundant (at genus level). **E** Mantel test assessing associations between10 key differential metabolites and key bacteria abundant (at genus level). Positive and negative correlations are shown by the red and blue matrices, respectively. Dashed lines indicate non-significant coefficients, and solid lines indicate significant coefficients. CON represents hens fed with basal diet; GL represents hens fed with a basic diet supplemented with 100 mg/kg glycyrrhizin

To investigate the effects of dietary GL on the intestinal microflora of aged breeder hens, 16S rRNA gene sequencing was performed (Fig. 7). Alpha diversity indices showed that the diversity index (Shannon) was significantly lower in the GL group than in the CON group (*P* < 0.05, Fig. 7A). However, no significant differences were observed in the Chao1, dominance, or observed_features indices between the two groups (*P* > 0.05). These results indicate that GL significantly altered the structure and composition of the gut microbiota. Venn diagram analysis revealed that 506 OTUs overlapped between groups, with 1,168 unique OTUs in the CON group and 604 in the GL group (Fig. 7B). Principal coordinates analysis (PCoA) further revealed significant separation between the microbial communities of the CON and GL groups (Fig. 7C). The relative abundance of microbes was analyzed by bar plot at the phylum and genus levels (Fig. 7D and E). At the phylum level, dietary GL significantly increased the relative abundance of Firmicutes and decreased the relative abundance of Bacteroidota in the ileal content (*P* < 0.05, Table 5). At the genus level, GL treatment significantly increased the relative abundance of *Lactobacillus* and *Limosilactobacillus*, and decreased the relative abundance of *Bacteroides*, *Rikenellaceae_RC9_gut_group* and *[Ruminococcus]_torques_group* (*P* < 0.05). The enriched taxa in the ileal microbiota of aged hens were presented in the cladogram (Fig. 7F). LEfSe analysis was employed to identify distinctive bacterial taxa between groups (*P* < 0.05, LDA score > 4) (Fig. 8A and B). The result indicated that GL treatment had a higher relative abundance of Firmicutes, Bacilli, Lactobacillales, Lactobacillaceae, and *Lactobacillus*. In contrast, the relative abundance of Bacteroidota, *Bacteroides*, and *Bacteroides_barnesiae* were significantly enriched in the CON group. Collectively, 16S rRNA gene sequencing of ileal content revealed that dietary GL improved the gut microbiota composition in aged hens.

**Table 5:** Effects of dietary glycyrrhizin supplementation on the relative abundance of bacteria at phylum and genus levels in ileal content

Items	Groups		*P*-value
	CON	GL	
Phylum			
Firmicutes	72.4 ± 12.5	95.3 ± 3.7*	0.005
Bacteroidota	17.2 ± 13.4	0.77 ± 1.0*	0.003
Actinobacteriota	3.94 ± 1.64	2.37 ± 1.36	0.103
Genus			
*Lactobacillus*	16.3 ± 12.0	52.6 ± 13.5*	0.001
*Ligilactobacillus*	17.3 ± 18.6	10.3 ± 4.8	0.426
*Romboutsia*	0.93 ± 0.59	8.20 ± 3.2	0.078
*Bacteroides*	6.05 ± 2.33	0.27 ± 0.16*	0.033
*Enterococcus*	1.52 ± 0.79	1.94 ± 1.41	0.803
*Rikenellaceae_RC9_gut_group*	3.39 ± 1.18	0.09 ± 0.04*	0.039
*[Ruminococcus]_torques_group*	2.76 ± 0.92	0.31 ± 0.13*	0.044
*Limosilactobacillus*	0.88 ± 0.21	2.65 ± 0.65*	0.029
*Rothia*	1.11 ± 0.67	0.80 ± 0.27	0.681
*Intestinimonas*	0.96 ± 0.56	0.03 ± 0.05	0.128

CON represents hens fed with basal diet; GL represents hens fed with a basic diet supplemented with 100 mg/kg glycyrrhizin. Data are expressed as mean ± SEM (*n* = 6). Asterisks indicate significant differences between groups (*P* < 0.05)

### Predicted function of intestinal microbiota in aged hens

The composition of the intestinal microbiota is closely linked to intestinal health and systemic metabolic disorders, particularly intestinal inflammation and obesity diseases. To investigate the functional alterations in the intestinal microbiota following dietary GL supplementation in aged hens, we performed Tax4Fun analysis to predict functional composition profiles (Fig. 8C). The results showed that 24 KEGG pathways were significantly enriched in the GL group compared with the CON group (*P* < 0.05). These enriched pathways included transporters and ABC transporters, as well as glycerolipid and sphingolipid metabolism, which are associated with gut homeostasis and the optimisation of energy metabolism. These changes appeared to be primarily associated with alterations in the abundance of *Lactobacillus* and *Bacteroides*.

To further explore potential relationships between serum biochemical parameters, 10 key differential metabolites, and the key intestinal bacteria in aged hens, Mantel test analysis was conducted (Fig. 8D and E). Notably, the abundance of *Lactobacillus* showed a significant negative correlation with hepatic levels of nicotinuric acid, glycodeoxycholic acid (hydrate), quinolinic acid, and S-(methyl) glutathione, but a positive correlation with spermine, taurocholic acid and tauroursodeoxycholic acid (*P* < 0.05). The abundance of *Bacteroides* was positively correlated with serum T-CHO and hepatic quinolinic acid, and negatively correlated with hepatic taurocholic acid (*P* < 0.05).

## Discussion

The rapid decline in reproductive performance of aged hens has always been a key concern in poultry production. Especially with the extension of the laying cycle, the egg-laying rate, egg quality and hatching rate typically decrease substantially [8, 42]. Recently, numerous studies have reported that dietary natural plant extract can improve the laying performance of aged breeder hens [2–4, 8]. These beneficial effects are thought to be mainly mediated via improving the function of the gut-liver axis and liver-ovarian axis in aged hens [17, 43]. This study demonstrates for the first time that dietary supplementation with 100 mg/kg of GL improves the reproductive performance of aged breeders, manifested as increased egg production and improved egg quality and higher hatching performance. These improvements are associated with GL’s ability to modulate gut microbial composition, improve liver lipid metabolism and alleviate ovarian ageing in aged breeder hens. Our findings comprehensively and systematically evaluate the effects of GL on aged hens and highlight its potential as a novel feed additive for maintaining the reproductive health of poultry.

A complex microbiota ecosystem exists in the intestinal tract of hens and plays vital roles in indigestion, absorption, and immune functions [44]. Previous studies have confirmed that gut microbiota composition and stability critically influence the reproductive performance of laying hens [17, 45]. With aging, long-term and high-intensity production compromises gut health in hens, leading to immune dysregulation and microbiota disruption, which ultimately impairs productivity [45]. Joat et al. [16] reported a gradual decline in the relative abundance of Firmicutes and an increase in Bacteroidete*s* from week 46 to 58 in laying hens, ultimately resulting in Bacteroidetes replacing Firmicutes as the dominant phylum in late-lay hens. In the present study, dietary GL supplementation reversed the age-related shifts in Firmicutes and Bacteroidota abundances. Meanwhile, GL treatment significantly increased the relative abundance of *Lactobacillus* but decreased *Bacteroides* at the genus level. Consistent with our results, dietary liquorice root powder, whose primary bioactive component is GL, has been shown to promote *Lactobacillus* proliferation in chickens in a dose-dependent manner [46]. Notably, although gut microbiota diversity decreased following GL intervention, this may reflect a compositional shift toward a beneficial microbial structure, characterized by increased abundances of beneficial bacteria (e.g., *Lactobacillus* and *Limosilactobacillus*) and decreased harmful taxa (e.g., *Rikenellaceae_RC9_gut_group* and *[Ruminococcus]_torques_group*). It is well known that there is a close correlation between intestinal microorganisms and the metabolic ability of the host [45]. Here, functional prediction via Tax4Fun revealed that dietary GL significantly enriched pathways related to transporters and ABC transporters, which are known to be involved in substrate transport and detoxification [47]. These findings suggest that GL improves intestinal health in aging hens by modulating the gut microbial structure. However, the underlying mechanism of change in intestinal function still requires further investigation. Previous studies have shown that gut microbes are closely related to metabolic disorders [48]. Gao et al. [49] demonstrated that reducing Bacteroidota abundance alleviated lipid metabolism disorders in broilers fed a high-fat diet. Similarly, we observed a positive correlation between *Bacteroides* abundance and serum T-CHO levels in this study. This indicates that GL may play potential regulatory roles in improving lipid metabolism in aged hens by increasing the abundance of *Lactobacillus* and decreasing the abundance of *Bacteroidetes*.

The liver is a central organ for lipid and protein metabolism in laying hens, directly influencing egg-laying performance. However, prolonged high-intensity laying in caged aged hens often leads to dyslipidemia and disordered hepatic lipid metabolism, characterized by increased abdominal fat deposition, hepatic steatosis, and elevated serum T-CHO and TG [3]. Numerous studies have confirmed the protective effects of GL on liver function and abnormal liver lipid metabolism, particularly in cases of non-alcoholic fatty liver disease [34, 50, 51]. In this study, GL treatment had a decreasing trend in abdominal fat mass and relieved the lipid vacuoles change of the liver of aged broiler hens, suggesting its regulatory role in lipid metabolism during the late laying phase. Simultaneously, the reduction trend in levels of metabolic markers, as well as serum TG and liver TG, and T-CHO, was also confirmed in this regard. Fatty acid synthase (FAS) activity has been reported to positively correlate with hepatic fat content in poultry [43]. To comprehensively analyze the effects of GL on liver lipid metabolism, the genes related to lipid synthesis were investigated in this study. Acetyl-CoA carboxylase (*ACC*) and *FAS* are the key regulatory enzymes of fatty acid synthesis in the liver, which are regulated by the nuclear transcription factor *SREBP-1c* [52]. In the present study, GL downregulated hepatic *FAS* and *ACC* mRNA expression in aged hens. Peroxisome proliferator-activated receptors α and γ (PPARα and PPARγ) are major regulators of hepatic lipid metabolism [53], and carnitine palmitoyltransferase-1 (CPT-1) is responsible for transporting fatty acids into mitochondria for β-oxidation [54]. We found that dietary GL supplementation upregulated the expression of *PPARα* and *CPT-1*, thereby enhancing hepatic fatty acid β-oxidation of aged hens. Consistent with our findings, previous studies demonstrated that GL promotes fatty acid β-oxidation by upregulating *PPARα*, *CPT1α*, and *ACADS* expression, while improving triglyceride metabolism in mouse models [55]. These findings indicate that GL reduces fat accumulation in the livers of aged hens by suppressing fatty acid synthesis and enhancing fatty acid β-oxidation. In order to explore the causes of changes in hepatic lipid metabolism, we further performed untargeted metabolomics analysis. Of note, the significantly altered metabolic pathways primarily included beta-alanine metabolism, nicotinate and nicotinamide metabolism, and taurine and hypotaurine metabolism, which play crucial roles in energy, bile acid, and lipid metabolism [56–58]. Previous studies have shown that GL can mitigate liver injury and reverse disturbances in the bile acid cycle [34]. In this study, GL supplementation significantly increased the hepatic levels of taurocholic acid, tauroursodeoxycholic acid, taurodeoxycholic acid (sodium salt), while decreasing the level of glycodeoxycholic acid (hydrate). It has been reported that *Bacteroides*, *Lactobacillus* and other gut microorganisms are involved in intestinal bile acid metabolism [59]. Interestingly, we found that *Lactobacillus* abundance was positively correlated with hepatic taurocholic acid and tauroursodeoxycholic acid, whereas *Bacteroides* was negatively correlated with hepatic taurocholic acid. Furthermore, as signaling molecules mediating gut-liver crosstalk, bile acids transcriptionally upregulate *PPARα* expression via FXR binding to its promoter [60]. These results suggest that GL may enhance fatty acid β-oxidation in aged hens, potentially by increasing bile acid levels in the enterohepatic circulation, though further validation is required. Collectively, we demonstrate for the first time that dietary GL modulates hepatic metabolic profiles in aged hens, potentially regulates the bile acid pathway and beta-alanine metabolism.

One of the main reasons for the decline in egg production in aged hens is the reduction in yolk synthesis and accumulation [8]. In poultry, egg yolk precursors are mainly synthesised in the liver and transported to the developing yolk follicles via the bloodstream [3]. Numerous animal studies have confirmed the benefits of enhanced hepatic yolk precursor synthesis for improving egg production in the late phase of laying hens [2, 43, 61]. In this study, dietary GL supplementation improved yolk precursor (vitellogenin, VTG) synthesis in the liver of aged breeder hens. Meanwhile, we found that small yellow follicles exhibited a significant positive correlation with hepatic VTG contents and a negative correlation with TG levels. This suggests that the improvement of egg production induced by GL is primarily attributed to enhanced yolk precursor synthesis, which promotes the development of small yolk follicles. Vitellogenin, a principal precursor of egg yolk proteins, exists in three forms: VTG-I, VTG-II, and VTG-III. Among these, VTG-II is the predominant form synthesized in hens [62]. Apolipoprotein B (APOB) and APOV1 are specific avian lipoproteins synthesized in the liver, which primarily bind to very low-density lipoprotein (VLDL) particles to facilitate the transport of yolk precursors [43]. In this study, GL treatment upregulated the expression of *VTG-II* and *APOV1* in the liver. The above results intensely indicate that GL improved hepatic lipid metabolism, which enhanced the function of yolk precursor synthesis in the liver. Whereas enhanced VTG precursor synthesis further improved follicular development and maturation in aged hens.

As the central organ of the reproductive system, ovarian senescence drives reproductive decline, characterized by a marked reduction in both the quantity and quality of eggs [4]. Declining reproductive hormone levels are regarded as one key cause of impaired reproductive performance in aged hens [4, 6]. Numerous studies have reported significantly lower serum estrogen and progesterone concentrations in laying hens during the late stages of egg production compared to the peak production phase [6, 63, 64]. This decline is often attributed to age-related ovarian oxidative damage, which inevitably disturbs follicular dysfunction and impaired steroidogenesis [4]. GL has been confirmed to possess potential antioxidative stress activity in vivo [65], primarily through its antioxidant effects through its strong capacity to scavenge ·OH [66]. Herein, the improvement of antioxidant status was also detected, with dietary 100 mg/kg GL increased T-AOC and reduced MDA levels in the ovary and serum of aged hens. In agreement, similarly study demonstrated that dietary GL at 50–150 mg/kg enhances the antioxidant capacity of the chicken's glandular stomach and alleviates tissue damage induced by zearalenone ingestion [38]. Spermine, a polyamine, participates in various biological processes including redox homeostasis and amino acid and lipid metabolism [67]. The formation of S-(methyl) glutathione through glutathione methylation reduces the availability of glutathione [68]. In this study, the increased hepatic spermine and decreased S-(methyl) glutathione levels also confirmed that dietary GL improved the antioxidant capacity in aged hens. It is commonly accepted that P4, produced by the granulosa cells of the pre-ovulatory follicle, regulates follicular development and promotes yolk deposition [69]. A recent study found that GL effectively alleviates ovarian dysfunction in a mouse model of polycystic ovary syndrome [70]. In poultry, *CYP11A1*, *CYP17A1*, *CYP19A1*, *3β-HSD* and *17β-HSD* are the major members of steroid hormone synthase in ovary [69]. Within mitochondria, *CYP11A1* converts cholesterol to pregnenolone, which is subsequently converted to progesterone by *3β-HSD* [71]*.* In agreement, dietary GL treatment up-regulated the ovarian expression of *LHR*, *CYP11A1*, and *3β-HSD*, thereby enhancing progesterone synthesis and potentially improving yolk precursor deposition in developing follicles. This effect aligns with the observed increase in the number of grade follicles and small yellow follicles. Notably, serum E2 levels remained unaltered in aged breeder hens following GL treatment, with invariant ovarian *CYP17A1* and *CYP19A1* mRNA expression. This may be related to the dose and duration of treatment in this study. However, further studies are required to determine the specific mechanisms of GL on the steroidogenesis of aged hens. Collectively, these results suggest that GL may help enhance follicular development by increasing serum progesterone levels and promoting yolk precursor deposition, thereby improving ovarian function in aging hens. In addition, it has been reported that the quality of breeder eggs, including albumen height and Haugh unit values, is closely associated with hatchability performance [72]. In the present study, dietary GL increased albumen height and Haugh unit in aged hens, which may contribute to improved hatchability of fertilized eggs. Overall, dietary GL supplementation improved egg quality, egg production, and hatchability of breeder eggs, thereby enhancing reproductive performance in hens during the late-laying stage.

## Conclusion

In summary, dietary supplementation with 100 mg/kg glycyrrhizin improved the egg production rate, egg quality, and hatchability in aged broiler breeder hens by modulating gut microbial composition, enhancing hepatic lipid metabolism and improving ovarian senescence. Specifically, GL supplementation enhanced hepatic yolk precursor (VTG) synthesis and ameliorated lipid metabolism through promoting β-oxidation and reversing age-related abundance changes in *Lactobacillus* and *Bacteroides*. Concurrently, GL supplementation promoted the development of grade follicles by enhancing ovarian antioxidant capacity and progesterone synthesis, along with increasing hepatic VTG synthesis. Our study revealed that GL supplementation can be used as a potential strategy to improve the reproductive health of breeder hens during the late laying period.

## Supplementary Information

Additional file 1: Table S1. Incubation parameters for chicken modes. Table S2. Primer sequences used in the present study. Table S3. Effect of dietary glycyrrhizin supplementation on the relative organs of aged hens.

## Data Availability

All data generated or analyzed during this study are available from the corresponding author on reasonable request.

## Abbreviations

3/17β-HSD: 3/17β-Hydroxysteroid dehydrogenase
ACC: Acetyl-CoA carboxylase
APOB: Apolipoprotein B
APOV1: Apovitellenin 1
ALB: Albumin
ALP: Alkaline phosphatase
ALT: Alanine aminotransferase
AST: Aspartate aminotransferase
CAT: Catalase
CPT-I: Carnitine palmitoyltransferase-I
CYP11/17/19A1: Cytochrome P450 family 11/17/19 subfamily A member 1
ERα/β: Estrogen receptor alpha/beta
GAPDH: Glyceraldehyde-3-phosphate dehydrogenase
GL: Glycyrrhizin
GSEA: Geneset enrichment analysis
HDL-C: High density lipoprotein cholesterol
LDL-C: Low density lipoprotein cholesterol
MDA: Malondialdehyde
OPLS-DA: Orthogonal partial least squares discriminant analysis
PCA: Principal component analysis
PPARα/γ: Peroxisome proliferator-activated receptor alpha/gamma
PR: Progesterone receptor
SREBP-1c: Sterol regulatory element-binding protein 1c
T-AOC: Total antioxidant capacity
T-CHO: Total cholesterol
T-SOD: Total superoxide dismutase
TG: Triglyceride
TP: Total protein
UA: Uric acid
VTG: Vitellogenin
