# Supplementary material for: Dietary glycyrrhizin enhances reproductive performance by improving intestinal microbiota, liver lipid metabolism and ovarian senescence in aged breeder hens
Source: J Anim Sci Biotechnol. 2025 Nov 20;16:156. doi: 10.1186/s40104-025-01288-5 (PMC12632042; doi:10.1186/s40104-025-01288-5)
Supplement: Supplementary file 1 — Additional file 1: Table S1. Incubation parameters for chicken modes. Table S2. Primer sequences used in the present study. Table S3. Effect of dietary glycyrrhizin supplementation on the relative organs of aged hens. [file 40104_2025_1288_MOESM1_ESM.docx]

Table S1. Incubation parameters for chicken modes.

| Incubation period (days) | 1-6 | 7-12 | 13-18 | After 19 |
| --- | --- | --- | --- | --- |
| Temperature, ℃ | 38.0 | 37.8 | 37.6 | 37.2 |
| Humidity, RH | 60% | 55% | 60% | 70% |
| Air exchange frequency, hour/time | 2（15） | 2（20） | 2（25） | 2（30） |
| Egg turning frequency, hour/angle | 1.5（180） | 1.5（180） | 1.5（180） | No |

Table S2. Primer sequences used in the present study.

| Gene | GeneBank ID | Primer sequence (5′→3′) | Product size (bp） |
| --- | --- | --- | --- |
| *GAPDH* | NM_203405 | F: GGTGAAAGTCGGAGTCAACGG | 244 |
|  |  | R: CGATGAAGGGATCATTGATGGC |  |
| *ERα* | NM_205183.2 | F: TGTGCTGTGTGCAACGACTA | 167 |
|  |  | R: CAGGCCTGGCAACTCTTTCT |  |
| *ERβ* | NM_204794.2 | F: CGGGCGTGGTGACATTAAAC | 192 |
|  |  | R: CCAGGATGAAGGGTGTGCAA |  |
| *LHR* | NM_204936.1 | F: GCAACGAATCGCTGACACTC | 141 |
|  |  | R: CTCTCAGGGCATCGTTGTGT |  |
| *PR* | NM_205262.1 | F: AGGAAGAACTGTCCAGCGTG | 233 |
|  |  | R: GCTCAATGCCTCGCAAAACA |  |
| *CYP11A1* | NM_001001756.1 | F: CATCTACCTGAGACTGCGGC | 134 |
|  |  | R: CTCCCCCATGGCGTAAGAAG |  |
| *CYP17A1* | NM_001001901.2 | F: CCCTACCTGGAGGCTACCAT | 145 |
|  |  | R: CGGACCAGAGGTTGATGACC |  |
| *CYP19A1* | NM_001001761.3 | F: GGCCTCCAGCAGGTTGAAAG | 214 |
|  |  | R: ATAGGCACTGTGGCAACTGG |  |
| *3β-HSD* | NM_205118.1 | F: TTTGTTTAGCACTGAGGCAAGAG | 255 |
|  |  | R: AGTCTTGCCCTGGAACTTGC |  |
| *17β-HSD* | NM_204837.1 | F: CCGCAGGTTCAAAGTGTTCG | 183 |
|  |  | R: TTGCACACCAAGACGTCCG |  |
| *FAS* | NM205155 | F: GCAGCTTCGGTGCCTGTGGTT | 119 |
|  |  | R: GCTGCTTGGCCCACACCTCC |  |
| *ACC* | NM205505 | F: AACGAGTCGGGCTACTACCT | 119 |
|  |  | R: ATCAGCATCCCGTGAAGTGG |  |
| *SREBP-1c* | AY029224 | F: TCACCGCTTCTTCGTGGAC | 144 |
|  |  | R: CTGAAGGTACTCCAACGCATC |  |
| *PPARα* | NM001001464 | F: TGTGGAGATCGTCCTGGTCT | 103 |
|  |  | R: CGTCAGGATGGTTGGTTTGC |  |
| *PPARγ* | NM_001001460.2 | F: TGACAGCGCCAGAGATTACA | 93 |
|  |  | R: CATCCATCGCAGACAGATCCA |  |
| *CPT-I* | NM001012898 | F: TCGTCTTGCCATGACTGGTG | 143 |
|  |  | R: GCTGTGGTGTCTGACTCGTT |  |
| *APOB* | NM_001044633.1 | F: GCCGTTTGACTGGGAGTACA | 126 |
|  |  | R: TCTTCCCATTTCCTGGTGCC |  |
| *APOV1* | XM_015295934.1 | F: CAAATGGGGAAACAAAGCAGGA | 220 |
|  |  | R: CTTCAGGGACAGTGGTGCTA |  |
| *VTG-I* | NM_001004408.3 | F: CTTATGCACCTCTTGCGTGC | 96 |
|  |  | R: ATCCAAAAGGTAGCGCCTGT |  |
| *VTG-II* | NM_001031276.1 | F: TTGCAAGCTGATGAACACACAC | 192 |
|  |  | R: GATTGCTTCATCTGCCAGGTC |  |

F: represents forward, R: represents reverse.

Table S3. Effect of dietary glycyrrhizin supplementation on the relative organs of aged hens.

| Items | Groups | | *P*-value |
| --- | --- | --- | --- |
|  | CON | GL |  |
| Abdominal fat, g/kg | 31.3 ± 3.7 | 21.9 ± 2.7 | 0.066 |
| Liver, g/kg | 14.7 ± 0.6 | 15.9 ± 0.7 | 0.194 |
| Spleen, g/kg | 1.00 ± 0.07 | 0.88 ± 0.05 | 0.178 |
| Ovary, g/kg | 14.5 ± 0.5 | 15.6 ± 1.1 | 0.196 |

CON, represents hens fed with basal diet; GL, represents hens fed with a basic diet supplemented with 100 mg/kg glycyrrhizin. Mean value ± SEM is used to represent data (n = 8).
